# Silver nanoparticles in resin luting cements: Antibacterial and physiochemical properties 

**DOI:** 10.4317/jced.52983

**Published:** 2016-10-01

**Authors:** Ana-Paula-Rodrigues Magalhães, Francine-Couto-Lima Moreira, Denise-Ramos-Silveira Alves, Cyntia-Rodrigues-Araújo Estrela, Carlos Estrela, Marcus-Santos Carrião, Andris-Figueiroa Bakuzis, Lawrence-Gonzaga Lopes

**Affiliations:** 1 DDS, MS, Student, Department of Prevention and Oral Rehabilitation, Dentistry School, Federal University of Goiás, Goiânia, Goiás, Brazil; 2 DDS, MS, PhD, Student, Department of Prevention and Oral Rehabilitation, Dentistry School, Federal University of Goiás, Goiânia, Goiás, Brazil; 3 Physicists, MS, PhD, Student, Department of Prevention and Oral Rehabilitation, Dentistry School, Federal University of Goiás, Goiânia, Goiás, Brazil; 4 Physicists, MS, PhD, Department of Prevention and Oral Rehabilitation, Dentistry School, Federal University of Goiás, Goiânia, Goiás, Brazil

## Abstract

**Background:**

Silver has a long history of use in medicine as an antimicrobial and anti-inflammatory agent. Silver nanoparticles (NAg) offer the possibility to control the formation oral biofilms through the use of nanoparticles with biocidal, anti-adhesive, and delivery abilities. This study aims to evaluate the antibacterial effect of resin luting cements with and without NAg, and their influence on color, sorption and solubility.

**Material and Methods:**

NAg were incorporated to two dual-cured resin cements (RelyX ARC (RA) color A1 and RelyX U200 (RU) color A2) in two concentrations (0.05% and 0.07%, in weight), obtaining six experimental groups. Disc specimens (1x6mm) were obtained to verify the antibacterial effect against *Streptococcus mutans* in BHI broth after immersion for 1min, 5min, 1h, 6h, and 24h (n=3), through optical density readings. Specimens were evaluated for color changes after addition of NAg with a spectrophotometer (n=10). Sorption and solubility tests were also performed, considering storage in water or 75% ethanol for 28 days (n=5), according to ISO 4049:2010. Data were subjected to statistical analysis with ANOVA and Tukey (*p*=0.05).

**Results:**

The optical density of the culture broths indicated bacterial growth, with and without NAg. NAg produced significant color change on the resin cements, especially in RA. Solubility values were very low for all groups, while sorption values raised with NAg. The cements with NAg did not show antibacterial activity against *S. mutans*. They also showed perceptible color change and higher sorption than the materials without NAg.

**Conclusions:**

The resin luting cements with NAg addition did not show antibacterial activity against S*S. mutans*. They also showed perceptible color change and higher sorption than the materials without NAg.

** Key words:**Silver, resin cements, products with antimicrobial action, solubility, color perception tests.

## Introduction

Dental caries has been a serious health problem for centuries. The disease initiation and development involves acidogenic bacteria, including *Streptococcus mutans*, *Streptococcus sobrinus*, and *Lactobacillus spp* (1,2). Although the prevalence of primary caries has been on decline worldwide since early 1980s, secondary caries remains an unresolved problem in Dentistry (3). The use of antimicrobial agents should ideally prevent biofilm formation without markedly affecting the biological equilibrium within the oral cavity (1,4).

When applying indirect restorations, bacteria may still be present under the restoration when the tissue affected by caries is not fully removed or if there is microleakage present after cementing (5). This may cause an increase of bacterial colonies, especially *S. mutans*, under the restoration inducing secondary caries and particularly reducing its longevity (2,5). Thus, the effects of luting cements with anticariogenic properties on oral microorganisms have to be considered.

Silver has a long history of use in medicine as an antimicrobial and anti-inflammatory agent (2,6-9). It has been incorporated to several materials in Medicine and Dentistry in different forms: silver zeolites, ions, microparticles, and nanoparticles (3,9,10). Silver nanoparticles (NAg) are clusters of silver atoms that are insoluble and smaller than 100 nm in size (11-13). Their size is an important characteristic because smaller particles give rise to higher specific surface areas, and therefore reduce the particle concentration necessary for efficacy (11,14). NAg offers the possibility to control the formation of these and other oral biofilms through the use of nanoparticles with biocidal, anti-adhesive, and delivery abilities (1).

However, the addition of a material as NAg in dental composites has to be done in the lowest concentration of nanoparticles capable of maintaining sufficient antibacterial effect without adversely affecting other material properties, like color and mechanical properties (11,14,15). In spite of the importance of esthetic for todays Dentistry, there are few reports in the literature of the color changes caused by silver addition in dental materials (15-17). Fan *et al.* (15) reported that when developing an antimicrobial light-cured resin, as the concentration of NAg increased the degree of cure decreased. When composites are immersed in water, a rapid elution of the unreacted monomers takes place and simultaneously water is absorbed by the resin, occupying the holes left by the monomers and affecting adversely their mechanical properties (15). Therefore, the water sorption and solubility behavior of resin luting cements added by NAg is very important from the clinical point of view.

The resin luting cements are dual-cured materials, indicated for cementation of ceramic or porcelain restorations of considerable thicknesses, like crowns, onlays, inlays, and bridges, and widely applied in Dentistry (18). Considering the importance of the resin luting cements antibacterial activity for the indirect restorations longevity, this study aimed to evaluate the antibacterial effect of resin luting cements with NAg addition, and their influence on color, sorption and solubility. The null hypotheses were: [1] there is no difference in antibacterial effect of resin luting cements with or without the addition of NAg [2] there is no difference in the color obtained in luting cements added or not with NAg; [3] there is no difference in solubility for resin luting cements with or without NAg addition, and [4] there is no difference in sorption between luting cements with and without NAg.

## Material and Methods

Two dual-cured resin luting cements were tested in this study: one conventional (RelyX ARC, 3M ESPE, St. Paul, USA; color A1), one self-adhesive (RelyX U200, 3M ESPE, St. Paul, MN, USA; color A2) ( Table 1). Each material had NAg incorporated during manipulation. The materials were handled according to the guidelines of ISO 4049:2010 (19), in regards to specimens preparation.

**Table 1:**
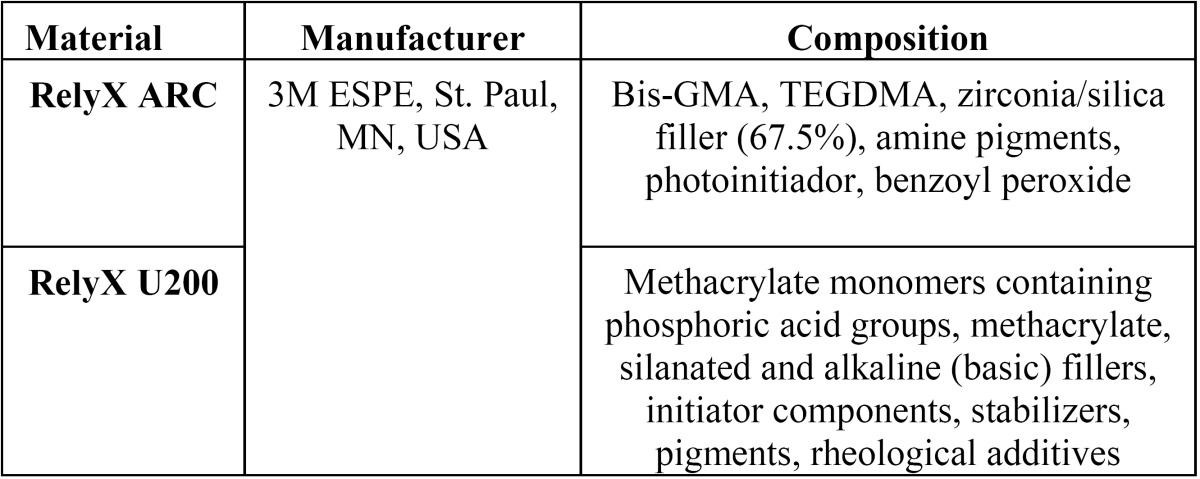
Resin cements used in this study and their composition.

-Silver nanoparticles production and test

**Figure 1:**
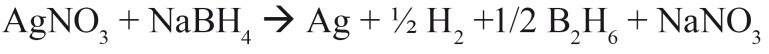
Reaction

The NAg were prepared in the Physics Institute of Federal University of Goiás (UFG), by reduction of silver nitrate (0.001 M) (Sigma-Aldrich, St. Louis, MO, USA) with sodium borohydride (0.002 M) (Sigma-Aldrich, St. Louis, MO, USA) in controlled low temperature and magnetic stirrer (TE 080, Techal, São José dos Campos, SP, Brazil). This method can be described by the reaction, (Fig. 1).

The solution obtained was added with 3 mL of a 0.75 M sodium chloride solution (Sigma-Aldrich, St. Louis, USA), then it went through a centrifugation process (Elektra, Laborline, Osasco, SP, Brazil) for 3000 rpm for 5 minutes, until the NAg was in the bottom of the flask. The water was removed and 1.5 mL of hydroxy-ethyl methacrylate (HEMA, Sigma-Aldrich, St. Louis, MO, USA) was added to the flask. This process was repeated twice to obtain a HEMA-NAg solution of 0.18% and three times to obtain a solution of 0.27% in NAg concentration. The final solutions were shaken in an ultrasonic apparatus (USC-2800, Unique, Indaiatuba, SP, Brazil) for 3 cycles of 10 minutes for complete dispersion of the metal in the material.

To confirm the antibacterial effect of this HEMA-NAg solution the Agar Diffusion Test was performed. Three Petri plates with 20 mL of Brain Heart Infusion (BHI, Difco Laboratories, Detroit, MI, USA) were inoculated with 0.1 mL of *S. mutans* (ATCC 25175). Three solutions were tested: HEMA without NAg, HEMA-NAg 0.18% and HEMA-NAg 0.27% in weight, with three sterile paper discs placed over the agar. The plates were incubated at 37oC for 48 h, after that the presence or absence of bacterial inhibition halos were observed. Halos were identified for all three solutions tested, being more evident for the groups with NAg added, confirming the inhibition of bacterial grow; while only a diffusion halo was observed for the HEMA without Nag solution, not representing inhibition.

-Specimens preparation

This study was performed in compliance with ISO 4049:2010 (19) standard specifications. Resin luting cements that belonged to the groups without NAg were manipulated according to manufacturer’s specifications (for at least 20 s), placed in a circle shaped stainless steel mold (1.0 mm thick, 6.0 mm diameter), and confined between two opposing polyester strips (3M ESPE, St. Paul, MN, USA). The ones with NAg addition had 10 μl of HEMA-NAg solution added prior to manipulation, and were then manipulated and placed in the same mold. All specimens were cured using a light-curing unit based on light emitting diodes (LED) (Emitter, Schuster, Santa Maria, Brazil) with continuous polymerization technique (600 mW/cm2, for 60 s). Light irradiance was checked before each photocure with a radiometer (Kerr Corporation, Orange, USA) to ensure consistency of light output.

The cements were divided in three groups each, a total of 6 groups: G1- RelyX ARC, G2- RelyX ARC with 0.05% (in weight) of NAg, G3- RelyX ARC with 0.07% NAg, G4- RelyX U200, G5- RelyX U200 with 0.05% NAg, and G6- RelyX U200 with 0.07% NAg.

-Antibacterial test

Standard strain of *Streptococcus mutans* (ATCC 25175) was obtained from American Type Culture Collection. The strain was inoculated into 7 mL of BHI broth (Difco Laboratories, Detroit, MI, USA) and incubated at 37˚C for 24 h. After the incubation period, the bacterial cells were resuspended in four test-tubes with 10 mL of BHI broth in a final concentration of approximately 3x108cells/ml in each tube in accordance to the turbidity standard 1 of McFarland scale.

One hundred and eight (108) specimens were used to the antibacterial test, based on previous studies. The specimens belonging to each group were stored in a Petri dish appropriately identified. Three specimens of each group were kept without contamination with the bacteria, the negative control. A quantity of 10 mL of bacterial suspension was added to each dish for contamination of the different groups specimens with *S. mutans*. Contamination was carried out at different time periods for each group: 1 min, 5 min, 1 h, 6 h, and 24 h (n = 3). Then, the specimens were placed in test tubes with 10 mL of deionized sterile water for washing with vortexing (Model AP 56, Phoenix, Araraquara, SP, Brazil) for 1 minute. In sequence, they were reconditioned to test tubes with sterile BHI broth and incubated for 48 hours at 37oC under 5% CO2 in a bacteriological incubator (model B2CBE, Deleo, Porto Alegre, RS, Brazil). After that, 2 mL of broth sample were collected from each tube and their optical density (turbidity) were read by UV spectrophotometer (UV Spectrophotometer Model 1600 New, Piracicaba, SP, Brazil) at λ = 600 nm, adopting as a standard the level 1 of McFarland scale, which corresponded to the absorbance of 0.137 nm after reset the device for BHI sterilized reading.

-Evaluation of color changes

Sixty specimens were used to evaluate the color change, being n=10 for each group, the same specimens used in the sorption and solubility tests. The measurement of the color of the specimens was performed with an Easyshade spectrophotometer (Vita, Bad Säckingen, Germany). For each specimen three readings were made using the parameters CIELab system (L* indicates lightness, a* represents the color saturation in red-green axis b* means color and saturation in blue-yellow axis). The reading for the determination of color parameters was always performed at the central point of the specimen in the same environment with the same lighting conditions, against a standard white background (Standard for 45o, 0o Reflectance and Color Garder Laboratory Inc., Annapolis, MD, USA).

**Figure 2:**
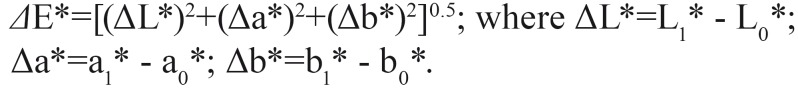
Formula.

The influence of NAg in color was directly expressed by ΔE* values that indicate the color difference between an initial condition collected on G1 and G4 specimens, and the other evaluated condition collected on G2 and G5 (ΔE1*) and also in G3 and G6 (ΔE2*) specimens, respectively. The ΔE* represents the total color change and was calculated from the formula: (Fig. 2).

-Sorption and solubility test

This test was done in compliance with ISO 4049:2010. (19) Sixty specimens, according to the ISO standard, were placed in opened glass bottles (Saint Gabain, São Paulo, SP, Brazil) and stored in a desiccator (Vidrolabor, São Paulo, SP, Brazil) containing freshly dried blue silica (Vetec, Rio de Janeiro, RJ, Brazil) in a model 002 CB oven (Fanem LTDA- modelo 002 CB, Guarulhos, SP, Brazil) at 37±1ºC for 22 hours. They were then removed, maintained at 23±1ºC for two hours, and then weighed in an analytical balance (Marte AY 220, Santa Rita do Sapucaí, MG, Brazil) accurate to 0.0001 g, and returned to the desiccator. The complete cycle was repeated until a constant mass (M1) was obtained, i.e., until the mass loss for each specimen was no more than 0.1 mg per 24 h cycle. Thereafter, the specimens were carefully placed back in their labeled bottles, and 15 mL of permeants, either deionized water or 75% ethanol, were added using manual pipettes (one for each permeant). The bottles were capped, brought back into the oven and kept at a temperature of 37ºC±1ºC for twenty-eight days.

After this time period, all the bottles were removed from the oven and kept at room temperature (23ºC±1ºC) for two hours. The specimens were removed from the bottles, washed in tap water for 15 seconds and left in a sterile bucket (Duflex, Rio de Janeiro, RJ, Brazil) for 1 minute. They were then weighed again to obtain M2. After weighting, the specimens were reconditioned in the desiccators until they reached a constant weight (M3) using the cycle described for M1.

The values for water sorption (Wsp) and solubility (Wsl) in micrograms per cubic millimeter were calculated using the following equations: Wsp=(M2-M3)/V; Ws1=(M1-M3)/V; V= πr2.h. Where: V is the volume, r is the radius, and h is the height. These measurements were obtained using a digital electronic caliper (Mitutoyo America, Tokyo, Japan), measuring the thickness of each specimen at four points and at its center, and the diameter in two points.

-Statistical analysis

Results obtained for all tests were described by mean and standard deviation. Due to the normal distribution of the variables (Kolmogorov-Smirnov and Shapiro-Wilk), ANOVA was performed to verify the effect of the different variables and Tukey test for multiple comparisons. Statistical analysis was carried out in software IBM SPSS Statistics 19.0 for Windows (SPSS Inc., Chi-cago, IL, USA), with a significance of 5% (*p*<0.05).

## Results

The results obtained for the optical density test are shown in  Table 2. Besides the control groups, that did not have contact with the bacteria, all groups showed optical density values greater than 0.137nm (standard 1 of McFarland scale). There were no statistically significant differences between the cements at different periods of contamination (*p*>0.05), except for G2 and G3 in 6h period. These groups showed statistically significant differences (*p*<0.05) between each other, being the 0.05% concentration the highest value.

**Table 2:**
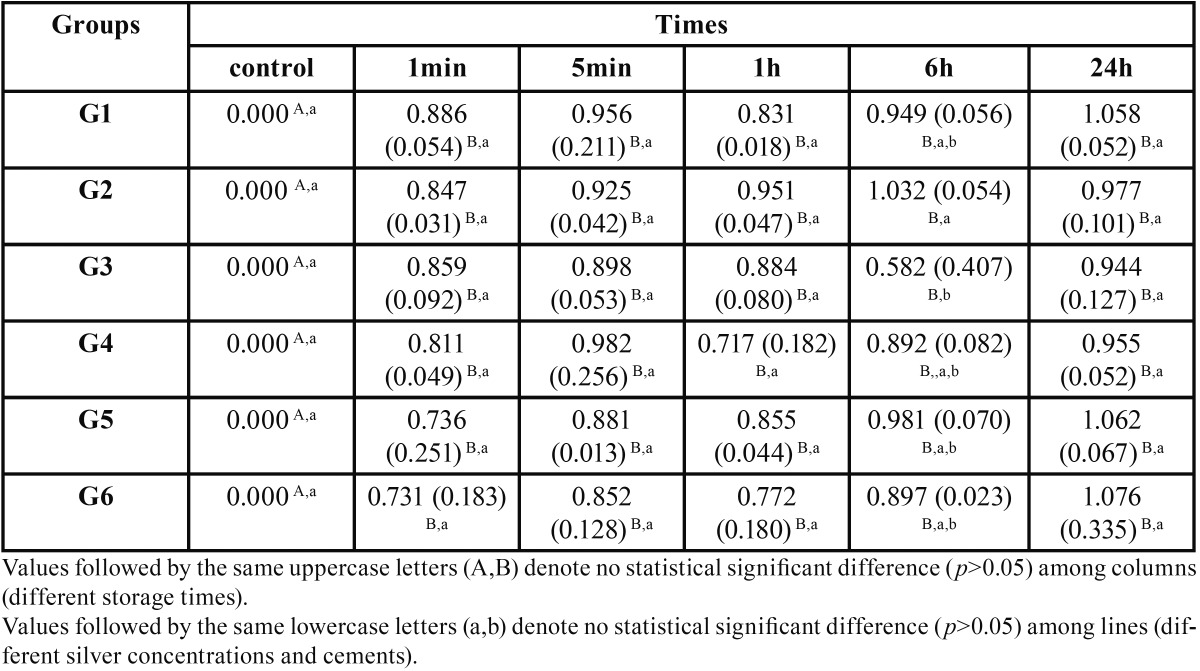
Values of mean and standard deviation (SD) for the optical density of the specimens culture broths contaminated, in nm.

The results obtained for the color evaluation are presented in  Table 3. After adding silver nanoparticles in their composition, both cements exhibited significant color changes. For the L* value, test groups showed statistical significant difference (*p*<0.05) from the control group. The a* value did not show significant difference between G1 and G2 (*p*>0.05), but were significant lower than G3 (*p*<0.05); G4, G5 and G6 showed significant changes in a* value (*p*<0.05), being G6 the highest. For b* there were significant differences between G1, G2 and G3 (*p*<0.05), and there was no difference between G4 and G6 (*p*>0.05), although they showed significant differences from G5 (*p*<0.05). Considering the color change (ΔE), the values observed for RelyX ARC were significantly higher (*p*<0.05), when compared to U200 with the same NAg content.

**Table 3:**
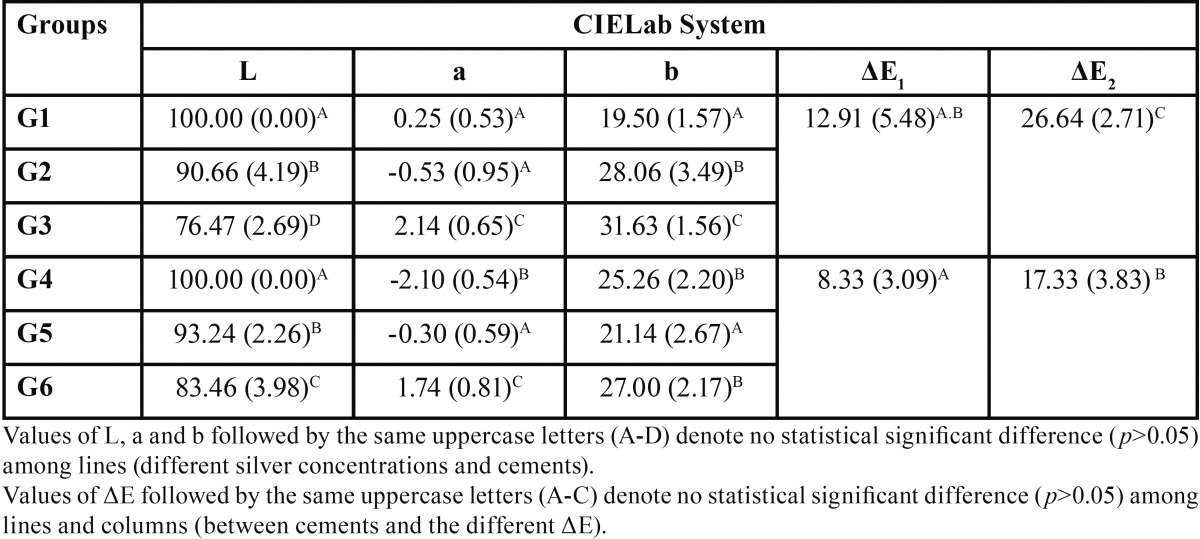
Values of mean and standard deviation (SD) for L*, a* and b* measured for each group and values of ΔE* calculated.

The results obtained for solubility and sorption are presented in  Table 4 and  Table 5, respectively. No statistical significant differences were observed for solubility among all the groups stored in water, independent of cement or NAg content (*p*>0.05). For the ones stored in ethanol, G5 was statistically different from the other groups (*p*<0.05), except from G6 (*p*>0.05). Among all other ethanol groups there were no significant statistical differences (*p*>0.05). Considering the solvents, no statistical difference was observed between specimens stored in water or ethanol (*p*>0.05), but for groups G5 and G6 (*p*<0.05) where ethanol storage led to higher solubility.

**Table 4:**
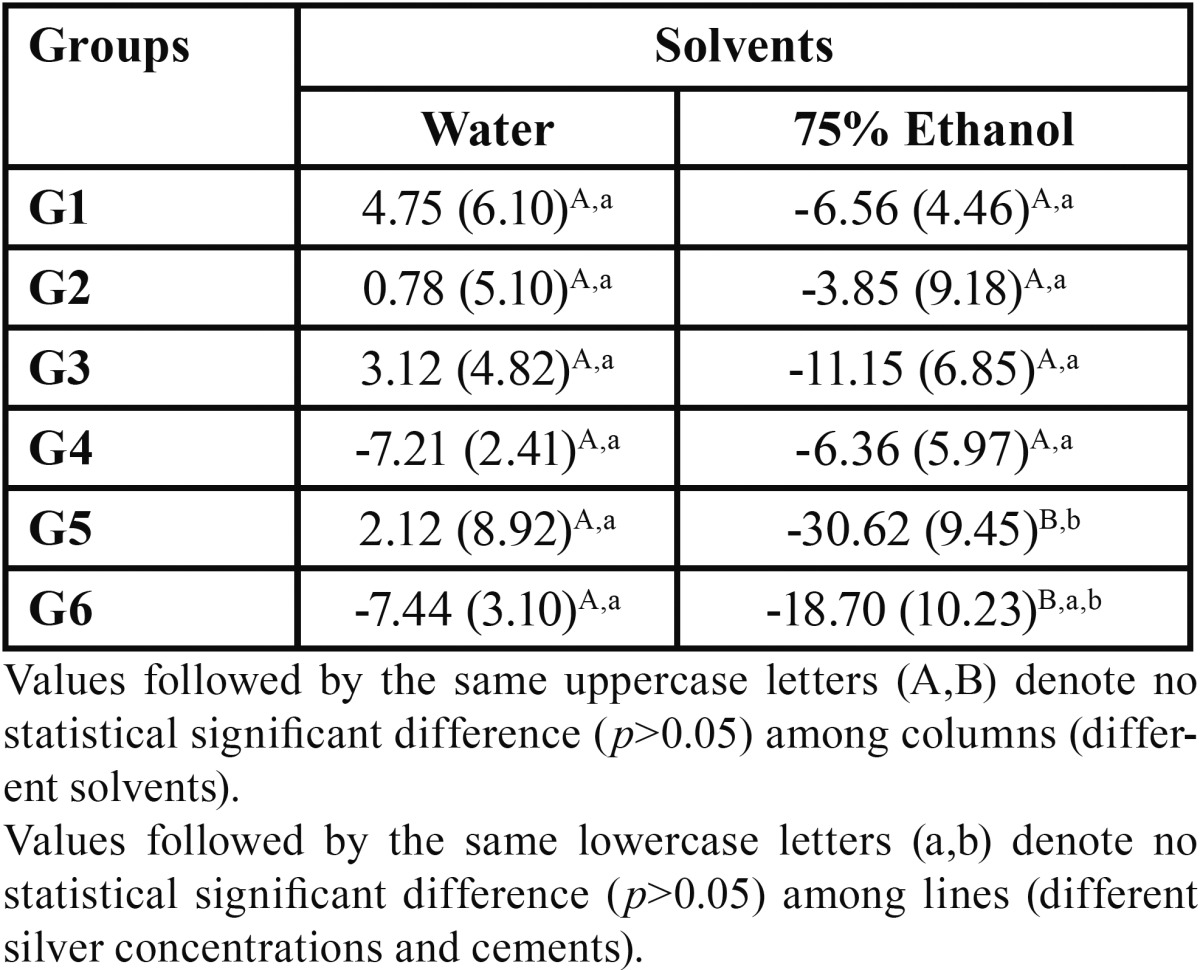
Values of mean and standard deviation (SD) for solubility found for the different cements and silver concentrations tested, in µg ∕ mm3.

**Table 5:**
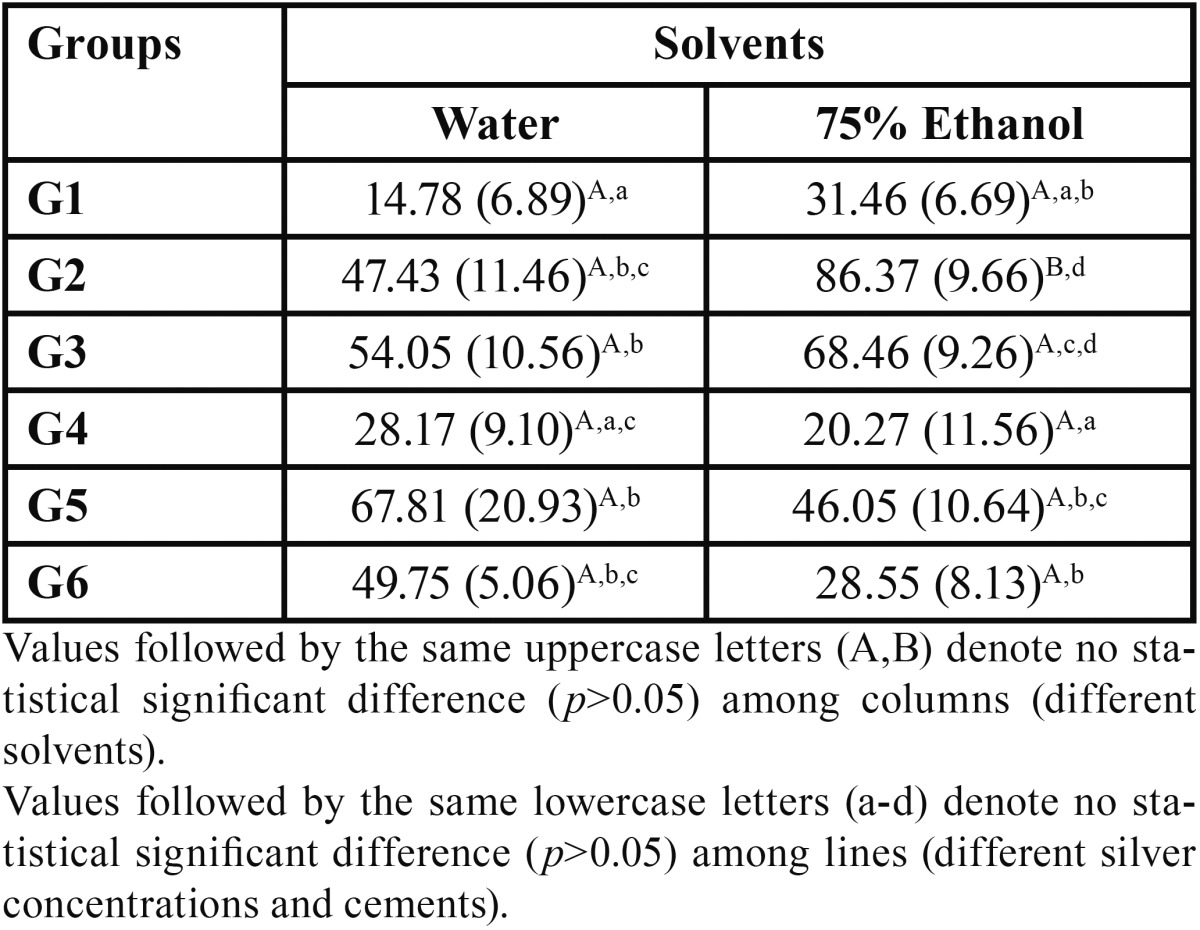
Values of mean and standard deviation (SD) for sorption found for the different cements and silver concentrations tested, in µg ∕ mm3.

For sorption in water, both control groups showed no statistical difference between each other (*p*>0.05), but were statistically different from all the other groups tested for the same cement (*p*<0.05), except for G4 and G6, that showed no statistical difference (*p*>0.05). There were no statistical difference also between G2 and G3; also for G5 and G6 in water (*p*<0.05). For the ethanol groups, the two control groups also showed no statistical difference from each other (*p*>0.05), but were statistically different from all the other groups tested for the same cement (*p*<0.05). G1 and G3 showed statistical significant differences (*p*<0.05), while G2 was similar only to G3 (*p*>0.05). G5 and G6 showed no significant difference between each other (*p*>0.05). Considering the solvents, there were no statistical significant differences between the groups stored in water and the ones in ethanol (*p*>0.05) but for G2, where ethanol storage led to higher sorption values (*p*<0.05).

## Discussion

NAg application in Medicine and Dentistry has been encouraged by the broad-spectrum antimicrobial effect in low concentrations, and the ability to not cause resistant bacterial strains to develop (1,11,13,15,16). The bactericidal mechanism of silver is only partially understood. One of the mechanisms is based on oxygen changing into active oxygen (ROS and hydroxyl radicals), and it causes structural damage in the bacteria (6,10,20). Other explanation is based on the release of silver ions that are biologically active and can interact with proteins, amino acid residues, enzymes, free anions, and receptors in the cell membranes; all these damage may cause the DNA to lose its ability to replicate (7-10). Other possible explanation that is being reported by recent studies is the direct contact of the particles with the cell wall, releasing silver ions in a very high concentration in a small area, killing the cell (6,20,21). This is important to explain the antibacterial activity of NAg entrapped in resin materials, as bone cement, denture basis and also composite materials for Dentistry, as resin luting cements.

The antimicrobial effect of silver against *S. mutans* has already been reported in literature (2-4,11,21). The HEMA-NAg, according to the Agar Diffusion Test, showed bactericidal effect on the target *S. mutans*, inhibiting bacterial growth. However the NAg incorporated to the resin cements did not present antibacterial effect against *S. mutans*, as bacterial growth was observed in all groups but the negative control, where no bacteria were seeded. According to Monteiro *et al.* (13) silver nanoparticles can have a limited use as biocidal materials in liquid systems because of their low colloidal stability, which is the situation observed in dentistry: silver has to be released from the restorative material and reach bacteria in liquid medium (saliva). Other authors have reported antibacterial effect of silver in very low concentrations, (2,10,11) however it is dependent on the surface energy: the lower the size of the particle the higher antibacterial effect it presents. With smaller particles is possible to have antibacterial effect in low concentrations, but the dispersion of silver in other materials is challenging, as NAg present a high propensity for aggregation, leading to lower surface energy and consequently, lower antibacterial effect (11). Thus, the low concentration of silver in the resin cement specimens (0.05%-0.07%), used in the present study, considering the whole volume of culture broth, may explain that finding. Also, as the NAg were trapped in the specimens resin, making the direct contact of silver with the bacteria minimal, also after polymerization probably few particles were eluted from the discs, leading to no or very small antibacterial effect. Considering this discussion, the first null hypothesis cannot be accepted.

One probable disadvantage for the use of silver in Dentistry that has been reported is the color, because of oxidation reaction (2,6,15-17,18,22). Cheng *et al.* (16) found that a dental composite with a mass fraction of silver higher than 0.042% showed a brownish color and lower mechanical strength. Nam *et al.* (20) reported color changes of denture base materials ranging from ΔE=15.6 to ΔE=28.6, which were considered unacceptable. Zhang *et al.* (22) found that adhesives added of 0.05-0.15% of silver showed no color change. ΔE values ≥ 3.3 are considered noticeable by a non-trained person and because of that, clinically unacceptable (23). In this study, significant color changes were found between the cements without NAg and the ones with NAg, especially for RelyX ARC, being the ΔE1 found for RelyX ARC (12.91) similar to the ΔE2 calculated for RelyX U200 (17.33). That was probably more evident for the first because its color was A1, while the RelyX U200 was A2. The cements were used in different colors because they do not have a match color between each other, but that allowed the observation that the lighter the resin cement color the higher probability of noticeable color change to occur. This finding leads to rejecting the second null hypothesis but it does not inhibit the use of NAg in these cements due to its applications, as most of the crowns, onlays, inlays, and bridges are not highly influenced by the background or cement color.

It is known that inadequate polymerization is usually associated with poor mechanical and biological properties because the unreacted monomers and fillers can be released by the resin based-materials in the aqueous environment, resulting in deterioration of the mechanical properties (24). A few studies have been made evaluating the influence of NAg addition in the polymerization process (8,15,25). Based on previous studies, Durner *et al.* (8) suggested that NAg can influence the amount of elutable substances from light hardened composite specimens, as the NAg interact even in low concentrations with the polymerization process through interaction with the light from the curing lamp and the photoinitiator system. Fan *et al.* (15) reported that NAg might form agglomerates around the polymer, preventing the cross-linking formation. The phenomena of sorption and solubility are closely related to the polymerization quality (26). In this study sorption and solubility were clearly influenced by NAg addition and concentration.

The third null hypothesis was accepted for water storage but partially rejected for ethanol storage, as there were significant differences in solubility between RelyX U200 groups with and without NAg. The solubility values calculated were mostly negative. This has been reported in previous studies (25,27,28) and is usually indicating that these materials were more susceptible to sorption, masking the solubility values. In accordance with these studies, it can be implicated that this does not mean that no solubility occurred, rather, that there was mass gain (27,29). Besides this, all solubility values are lower than the maximum value established by the ISO 4049:2010 (19) for polymer-based materials (7.5 μg/mm3).

Considering sorption, the lowest values observed were the groups without NAg, this is expected as the resin luting cement without NAg and manipulated as manufacturers recommendation is supposed to deliver the best properties, rejecting the fourth null hypothesis. The self-adhesive cements contain hydrophilic acidic monomers, such as phosphoric acid, that may result in higher solvent uptake, (28) thus higher sorption than the conventional cements, as showed in this study. The NAg content led to numerically different values, as the 0.07% concentration of NAg led to lower sorption than 0.05%. This can be explained by the two components added to these cements: NAg and HEMA. The increase in NAg content might contribute to a successive increase in the material hydrophobicity, leading to less water sorption when more NAg is present in the polymer matrix (4). Also NAg may act as filler particles, increasing the filler content of the cement and contributing to lower sorption (18). The hydrophobic nature of the monomers in the composites influence the sorption and solubility behavior of the cements, and more HEMA content is known to enhance water sorption and promote hydroscopic expansion of a material (18). The higher amount of HEMA in the 0.05% solution and the slightly lower content of NAg when compared to the 0.07% one might have led to these values of sorption observed in this study. Sorption was numerically higher for 75% ethanol when compared to water, this might be due to the enhanced ability of ethanol to penetrate and swell the crosslinked polymer network in comparison to water, promoting then an accelerated aging effect (18). Also, according to US FDA (United States Food and Drug Administration) guidelines (30), 75% ethanol-water is an important clinical simulating liquid, and considering that, it was used in this study.

To be accepted clinically, the new materials added with NAg must provide superior antimicrobial activity and display comparable physical properties when compared with conventional ones (6). Thus, the resin luting cement proposed by this study has to be further evaluated, especially for its toxicity, antibacterial and mechanical properties to be securely applied in clinical practice.

The resin luting cements with NAg addition did not show antibacterial effect against *S. mutans*. This resin cements with NAg showed perceptible color change and higher sorption than the materials without NAg.

## References

[1] Allaker RP (2010). The Use of Nanoparticles to Control Oral Biofilm Formation. J Dent Res.

[2] Kasraei S, Sami L, Hendi S, Alikhani MY, Rezaei-Soufi L, Khamverdi Z (2014). Antibacterial properties of composite resins incorporating silver and zinc oxide nanoparticles on Streptococcus mutans and Lactobacillus. Restor Dent Endod.

[3] Bürgers R, Eidt A, Frankenberger R, Rosentritt M, Schweikl H, Handel G (2009). The anti-adherence activity and bactericidal effect of microparticulate silver additives in composite resin materials. Arch Oral Biol.

[4] Das Neves PB, Agnelli JA, Kurachi C, de Souza CW (2014). Addition of silver nanoparticles to composite resin: effect on physical and bactericidal properties in vitro. Braz Dent J.

[5] Daugela P, Oziunas R, Zekonis G (2008). Antibacterial potential of contemporary dental luting cements. Stomatologija.

[6] Ahn SJ, Lee SJ, Kook JK, Lim BS (2009). Experimental antimicrobial orthodontic adhesives using nanofillers and silver nanoparticles. Dent Mater.

[7] Chaloupka K, Malam Y, Seifalian AM (2010). Nanosilver as a new generation of nanoproduct in biomedical applications. Trends Biotechnol.

[8] Durner J, Stojanovic A, Ebru U, Reinhard H, Franx-Xaver R (2011). Influence of silver nano-particles on monomer elution from light-cured composites. Dent Mater.

[9] Peng JJ, Botelho MG, Matinlinna JP (2012). Silver compounds used in dentistry for caries management: a review. J Dent.

[10] Saengmee-Anupharb S, Srikhirin T, Thaweboon B, Thaweboon S, Amornsakchai T, Dechkunakorn S (2013). Antimicrobial effects of silver zeolite, silver zirconium phosphate silicate and silver zirconium phosphate against oral microorganisms. Asian Pac J Trop Biomed.

[11] Cheng YJ, Zeiger DN, Howarter JA, Zhang X, Lin NJ (2011). In situ formation of silver nanoparticles in photocrosslinking polymers. J Biomed Mater Res B Appl Biomater.

[12] Kurek A, Grudniak AM, Kraczkiewictz-Dowjat A, Wolska KI (2011). New Antibacterial Therapeutics and Strategies. Pol J Microbiol.

[13] Monteiro DR, Gorup LF, Takamiya AS, Ruvollo-Filho AC, De Camargo ER, Barbosa DB (2009). The growing importance of materials that prevent microbial adhesion: antimicrobial effect of medical devices containing silver. Int J Antimicrob Agents.

[14] Lu Z, Rong K, Li J, Yang H, Chen R (2013). Size-dependent antibacterial activities of silver nanoparticles against oral anaerobic pathogenic bacteria. J Mater Sci Mater Med.

[15] Fan C, Chu L, Rawls HR, Norling BK, Cardenas HL, Whang K (2011). Development of an antimicrobial resin--a pilot study. Dent Mater.

[16] Cheng L, Weir MD, Xu HH, Antonucci JM, Lin NJ, Lin-Gibson S (2012). Effect of amorphous calcium phosphate and silver nanocomposites on dental plaque microcosm biofilms. J Biomed Mater Res B Appl Biomater.

[17] Uno M, Kurachi M, Wakamatsu N, Doi Y (2013). Effects of adding silver nanoparticles on the toughening of dental porcelain. J Prosthet Dent.

[18] Meşe A, Burrow MF, Tyas MJ (2008). Sorption and solubility of luting cements in different solutions. Dent Mater J.

[19] (2010). Dentistry – Polymer-based filling, restorative and luting materials.

[20] Nam KY, Lee CH, Lee CJ (2012). Antifungal and physical characteristics of modified denture base acrylic incorporated with silver nanoparticles. Gerodontology.

[21] Yoshida K, Tanagawa M, Matsumoto S, Yamada T, Atsuta M (1999). Antibacterial activity of resin composites with silver-containing materials. Eur J Oral Sci.

[22] Zhang K, Li F, Imazato S, Cheng L, Liu H, Arola DD (2013). Dual antibacterial agents of nano-silver and 12-methacryloyloxydodecylpyridinium bromide in dental adhesive to inhibit caries. J Biomed Mater Res B Appl Biomater.

[23] Johnston WM, Kao EC (1989). Assesment of appearance match by visual observation and clinical colorimetry. J Dent Res.

[24] Lopes LG, Magalhaes AP, Brandao NA, Carvalho AA, Moreira F do C, De Souza JB (2012). Effect of light source and solvent on the sorption and solubility of two dual-cured cements photocured through ceramic. Gen Dent.

[25] Chladek G, Kasperski J, Barszczewska-Rybarek I, Zmudzki J (2012). Sorption, solubility, bond strength and hardness of denture soft lining incorporated with silver nanoparticles. Int J Mol Sci.

[26] Ferracane JL (2006). Hygroscopic and hydrolytic effects in dental polymer networks. Dent Mater.

[27] Lopes LG, Jardim Filho A Da V, De Souza JB, Rabelo D, Franco EB, De Freitas GC (2009). Influence of pulse-delay curing on sorption and solubility of a composite resin. J Appl Oral Sci.

[28] Marghalani HY (2012). Sorption and solubility characteristics of self-adhesive resin cements. Dent Mater.

[29] Fabre HS, Fabre S, Cefaly DF, De Oliveira Carrilho MR, Garcia FC, Wang L (2007). Water sorption and solubility of dentin bonding agents light-cured with different light sources. J Dent.

[30] (2010). Guidance for industry: Enzyme preparations: Recommendations for submission of chemical and technological data for food additive petitions and GRAS notices.

